# Prevalence and management of post-transplant anemia in long-term follow-up of Chinese kidney transplant recipients: a single-center report

**DOI:** 10.1186/2047-783X-18-45

**Published:** 2013-11-15

**Authors:** Zhixian Wu, Junqi Guo, Lianming Liao, Weizhen Wu, Shunliang Yang, Jianming Tan

**Affiliations:** 1 Organ Transplant Institute, Fuzhou General Hospital, Xiamen University, 156 West Er’huan Road, Fuzhou 350025, China

**Keywords:** Anemia, Calculated creatinine clearance, Erythropoietin, Immunosuppressive, Iron deficiency

## Abstract

**Background:**

Post-transplant anemia (PTA) has long been a less-recognized complication in kidney transplant recipients, and its prevalence also tends to be underestimated. This study sought to evaluate the prevalence, management, and risk factors of PTA from a group of long-term follow-up Chinese kidney transplant recipients.

**Methods:**

One hundred and fifty-four adult kidney transplant recipients were followed up at Fuzhou General Hospital, China, and retrospectively studied.

**Results:**

PTA prevalence at transplant and at 5-yearly time points after transplantation were 45.5%, 10.7%, 9.6%, 14.8%, 13.5%, and 19.6%, respectively. Overall, 38.3% of patients had been anemic at least once during the follow-up period, and 42% of these patients had recurrent anemia. Correlation analysis indicated that hemoglobin levels were associated with graft function. No correlations between anemia and age, gender, immunosuppressive regimens, or antihypertensive agents were observed. Binary logistic regression analysis suggested that serum creatinine and blood urea nitrogen were associated with the diagnosis of anemia at 1 year post-transplant. At 5 years post-transplant, only serum creatinine concentrations correlated with anemia. Although iron drugs are frequently used, erythropoietin was rarely administered in those anemic patients suffering poor graft function that necessitated such therapies.

**Conclusions:**

The prevalence of PTA is noticeably high, and impaired kidney graft function seemed to be the major risk factor for anemia. There is an urgent need to improve current PTA management and to establish modified guidelines for this common complication in kidney transplant recipients.

## Background

In the first few weeks, and even months, after transplantation, it is relatively common for kidney transplant recipients to suffer from post-transplant anemia (PTA) due to blood loss from the surgical procedure and frequent blood sampling for repeated tests. Other factors, such as uremic toxins, iron deficiency, and erythropoietin resistance, may also account for the high incidence of PTA in this time period [1]. Despite the restoration of normal hemoglobin levels achieved in most patients with well-functioning grafts, we observed that a number of kidney transplant recipients developed anemia. Previous studies suggest that anemia can raise the risk of cardiovascular events in patients on hemodialysis, and cardiovascular diseases have been reported as the primary cause of death in kidney transplant recipients. It is therefore reasonable to suggest that anemia may harm the survival and quality of life of kidney transplant recipients through the same mechanism that affects dialysis patients [2]. If this is correct, treating anemia may offer improved clinical outcomes for kidney transplant recipients. However, data on the prevalence of PTA and its current management are neither adequate nor globally applicable, since different populations and diagnostic criteria have been used.

The aim of this study was to gain insight into the prevalence and risk factors of anemia from a group of long-term follow-up Chinese kidney transplant recipients, and to discuss the current management of this problem.

## Methods

One hundred and fifty-four adult deceased-donor kidney transplant recipients who underwent a kidney transplant between January 2005 and May 2008 were followed up at Fuzhou General Hospital, China, and enrolled if there was the potential for at least 5 years’ post-transplant follow-up. Of these patients, 14 died and 16 had graft loss prior to their 5-year post-transplant time point, and another six were lost to follow-up for various reasons. Demographic and clinical data of patients at transplant and in the subsequent 5 years during follow-up were retrieved (data being collected from office visit records of each patient at the date closest to that of transplantation, which was ± 1 month at the first and second year, and extended to ± 3 months for later years).

Patients were diagnosed as anemic according to the normative values established by the Chinese Society of Hematology [3], which are 12 g/dL for males and 11 g/dL for females. Anemic patients were further grouped as moderate or severe if their hemoglobin levels were below the lower limit of normal, and above 9.0 g/dL or below 9.0 g/dL, respectively. Anemia that developed 2 years or more after its first occurrence was defined as recurrent. Other clinical information, such as age, gender, weight, serum creatinine (Scr) levels, blood urea nitrogen (BUN) levels, calculated creatinine clearance (Ccr) using the Cockcroft–Gault equation, immunosuppressive regimens, and angiotensin-converting-enzyme inhibitors (ACEI) or angiotensin receptor blockers (ARB), were also recorded to assess their impact on hemoglobin levels and the diagnosis of PTA.

For analysis of the management of PTA, data regarding iron drugs and erythropoietin therapy were entered.

All patients gave signed informed consent. The study was approved by the Fuzhou General Hospital Institute Review Board (IRB00006161).

### Statistical analysis

Student *t*-tests or Mann–Whitney U tests were used for comparisons between two continuous variables. Chi-square tests were used for the detection of differences between proportions. Repeated measures ANOVA were used to detect differences among the means of variables of interest at each time point. Pearson’s or Spearman’s correlation coefficients were chosen to determine whether a relationship existed between two variables. All statistical analyses were performed using SPSS 11.0 software (IBM, Armonk, NY, USA). A *P* value of <0.05 was considered statistically significant.

## Results

Patients’ demographic data and the status of anemia at transplant and during follow-up are shown in Table 1. The mean duration before follow-up was 4.3 ± 1.7 years. Seventeen patients developed delayed graft function. At transplant, most patients (57.8%) received triple therapy consisting of cyclosporin A, mycophenolate mofetil (MMF), and prednisone, and 19.5% received a combination of cyclosporin A, azathioprine, and prednisone. Tacrolimus was not used as often as cyclosporin A at Fuzhou General Hospital, thus 20.8% of patients received a combination of tacrolimus, MMF, and prednisone, and a few patients (1.9%) received a combination of tacrolimus, azathioprine, and prednisone.

**Table 1:** Patients’ demographic data and anemia status at transplant and at each year of follow-up

**Year post-transplant**	**0**	**1**	**2**	**3**	**4**	**5**
Total (n)	154	140	136	128	126	118
Age (years)	38.1 ± 10.3	38.8 ± 10.4	39.9 ± 10.4	40.0 ± 9.9	41.1 ± 9.9	41.9 ± 10.0
Gender (male/female)	109/45	98/42	97/39	89/39	89/37	85/33
Immunosuppressive						
CsA + AZT + pred	30 (19.5%)	25 (17.9%)	20 (14.7%)	17 (13.3%)	16 (12.7%)	14 (11.9%)
CsA + MMF + pred	89 (57.8%)	81 (58.6%)	84 (61.8%)	80 (62.5%)	79 (62.7%)	73 (61.9%)
Tac + MMF + pred	32 (20.8%)	31 (22.1%)	30 (22.1%)	29 (22.7%)	29 (23.0%)	29 (24.6%)
Tac + AZT + pred	3 (1.9%)	2 (2%)	2 (1.5%)	2 (1.6%)	2 (1.6%)	2 (1.7%)
Mean Hb (g/dL)	11.5 ± 1.5	13.7 ± 1.9	13.7 ± 1.8	13.5 ± 1.9	13.4 ± 1.9	13.0 ± 2.0
Anemic						
n	70	15	13	19	17	23
Percentage	45.5%	10.7%	9.6%	14.8%	13.5%	19.6%

AZT, azathioprine; CsA, cyclosporin A; Hb, hemoglobin; MMF, mycophenolate mofetil; pred, prednisone; Tac, tacrolimus.

Patients at transplant had the highest prevalence (45.5%) of anemia for all recorded time points. In the proceeding 5 years, 38.3% of patients had at least one episode of anemia, and 42% of them experienced recurrent anemia. The prevalence of PTA was low in the early years; 10.7% and 9.6% at 1 and 2 years post-transplant, respectively. However, PTA increased over time as reflected by the prevalence of 19.6% at 5 years post-transplant. There was a significant (*P* <0.001) decrease in mean hemoglobin levels at 5 years post-transplant compared with those at 1, 2, 3, and 4 years; 30.4% of anemic patients at the 5-year time point had a hemoglobin level lower than 9 g/dL, but only 6.7% and 7.7% were observed at 1 and 2 years, respectively.

We selected the 1- and 5-year post-transplant variables and compared them for anemic and non-anemic patients (Table 2). Although generally considered as an important risk factor for poor graft function, delayed graft function did not lead to a significant difference in Ccr and the prevalence of anemia in this study at both time points. At 1 year post-transplant, there was a significant difference in Ccr between anemic and non-anemic patients. Sixty percent of anemic patients had a Ccr of less than 50 mL/min/1.73 m^2^; whereas only 12% of non-anemic patients showed diminished graft function. At 5 years post-transplant, anemic patients also suffered from impaired graft function, demonstrating higher BUN (*P* = 0.001) and Scr (*P* <0.001) levels, and lower Ccr (*P* = 0.001) than non-anemic patients. Of the seven patients with moderate anemia or worse, six patients (85.7%) showed a Ccr less than 50 mL/min/1.73 m^2^. However, poor renal excretory function did not have a causal relationship with anemia. There were two and four anemic patients who had Ccr higher than 75 mL/min/1.73 m^2^ at 1 and 5 years post-transplant, respectively. Some non-anemic patients did have suboptimal graft function.

**Table 2:** Comparisons between patients with or without anemia

	**1 year post-transplant**			**5 years post-transplant**		
	**Anemic**	**Non-anemic**	***P *value**	**Anemic**	**Non-anemic**	***P *value**
	**(n = 15)**	**(n = 125)**		**(n = 23)**	**(n = 95)**	
Age (years)	38 ± 11.8	38.9 ± 10.3	0.744	40.3 ± 9.1	42.3 ± 10.2	0.385
Gender (male/female)	10/5	88/37	0.766	16/7	69/26	0.769
BUN (mmol/L)	10.8 ± 6.1	6.9 ± 2.0	0.001	13.5 ± 8.1	7.1 ± 2.2	<0.001
Creatinine (mg/dL)	1.8 ± 0.6	1.3 ± 0.3	0.001	2.0 ± 0.8	1.3 ± 0.2	<0.001
Ccr						
Mean	51.1 ± 20.8	69.1 ± 17.3	<0.001	48.1 ± 21.9	65.8 ± 13.7	<0.001
>75 mL/min/1.73 m^2^	2 (13.3%)	40 (32%)	0.136	4 (17.4%)	25 (26.3%)	0.372
50 to 75 mL/min/1.73 m^2^	4 (26.7%)	70 (56%)	0.032	4 (17.4%)	61 (64.2%)	<0.001
<50 mL/min/1.73 m^2^	9 (60%)	15 (12%)	<0.001	15 (65.2%)	9 (9.5%)	<0.001
Immunosuppression						
Tac/CsA	3/12	30/95	0.73	3/20	28/67	0.108
MMF/AZT	12/3	110/15	0.382	21/2	81/14	0.448
ACEI or ARB (yes/no)	1/14	19/106	0.372	5/18	16/79	0.58
Iron medicine (yes/no)	11/4	12/113	<0.001	17/6	6/89	<0.001

ARB, angiotensin receptor blockers; ACEI, angiotensin-converting-enzyme inhibitors; AZT, azathioprine; BUN, blood urea nitrogen; Ccr, calculated creatinine clearance; CsA, cyclosporin A; MMF, mycophenolate mofetil; Tac, tacrolimus.

Cyclosporin A- or tacrolimus-based immunosuppression regimens, ACEI or ARB use, gender, and age (<55 versus >55 years of age) did not affect the prevalence of anemia at either 1 or 5 years post-transplant. Female patients seemed to have lower hemoglobin levels than males at both time points (female: 12.9 ± 1.88 g/dL, male: 13.4 ± 1.96 g/dL 1 year post-transplant; *P* = 0.002). At 1 year post-transplant, tacrolimus-based immunosuppressive treatment (*P* <0.001) and age <55 years (*P* = 0.025) were associated with better Ccr, while no significant correlation between Ccr and these factors were observed at 5 years post-transplant. There was no correlation between gender and ARB or ACEI use and Ccr at either 1 or 5 years post-transplant.

Patients receiving cyclosporin A, MMF, and prednisone, and patients receiving cyclosporin A, azathioprine, and prednisone were selected to determine which purine synthesis inhibitor was more likely associated with anemia. A similar prevalence of anemia was found in the earlier 4 years between these two groups of patients. Although a greater proportion of patients on MMF had anemia at 5 years post-transplant, the difference was insufficient to achieve statistical significance (MMF: 23.8% versus azathioprine: 7.1%; *P* = 0.109). Of the 12 patients who converted from azathioprine to MMF, seven patients (58.3%) developed anemia after conversion.

Iron therapy is frequently used at our center for the management of anemia. Overall, 87.1% of anemic patients were treated with iron drugs at transplant, and most anemic patients were still administered with medication including iron drugs at follow-up, which were prescribed for 73.3% and 73.9% of patients with anemia at 1 and 5 years post-transplant, respectively. Of the non-anemic patients, a small proportion (9.6% and 6.3% at 1 year and 5 years, respectively) also received iron therapy. However, erythropoietin was used much less often in kidney transplant recipients. None of the 15 patients with anemia at 1 year post-transplant received erythropoietin. At 5 years post-transplant, only one patient with moderate anemia (hemoglobin: 7.2 g/dL) received erythropoietin therapy.

Univariate correlation analyses indicated that hemoglobin levels were associated with BUN (1 year: *r* = −0.280, *P* = 0.001; 5 years: *r* = −0.210, *P* = 0.001) and Scr (1 year: *r* = −0.152, *P* = 0.008; 5 years: *r* = −0.146, *P* = 0.021).

Binary logistic regression analyses were performed for variables, including gender, original renal disease, dialysis duration, MMF levels, azathioprine doses, BUN, and Scr. Scr correlated with the diagnosis of anemia at both 1 and 5 years post-transplant, and BUN correlated with anemia only at 1 year post-transplant.

## Discussion

Despite its potential negative effects on kidney transplant recipients, PTA has only received moderate to mild attention. In the current study, we observed that anemia was a widespread problem: 38.3% of patients had at least one episode of anemia during the follow-up period, and 19.6% of patients were anemic at 5 years post-transplant.

Kidney graft function correlated with the development of anemia in this study, but interestingly, anemia was present in some patients whose kidney excretory function was good; conversely, it was absent in some cases with poor graft function. van Dullemen *et al*. [4] reported that erythropoietin production is lowered with the deterioration of kidney graft excretory function, while erythropoietin resistance becomes more apparent. This finding facilitates the understanding that anemic patients always have decreased Ccr and higher Scr values. However, identification of these patients suggests that other factors, rather than the excretory branch of the kidney graft, including inappropriate erythropoietin production resulting from delayed graft function or perhaps calcineurin inhibitor nephrotoxicity, may play a role in the pathogenesis of anemia. Since no data on serum erythropoietin assessment or graft biopsy were available in this study, these hypotheses remain to be determined. The effect of some much more common complications, such as poor nutritional status or folate/vitamin B_12_ deficiency, may also influence hemoglobin levels and the onset of anemia.

Overall, 38.3% of patients in this study had at least one episode of anemia during the follow-up period. Of these, 18 patients (42%) experienced recurrent anemia at later time points, suggesting susceptibility caused by some persistent clinical conditions in these patients.

The PTA prevalence observed at our center at 4 and 5 years post-transplant was lower when compared with that of previous reports [5]. The lower normative value used in this study is one of the possible explanations, but the more frequent administration of iron drugs for anemic patients is also likely to benefit the results. Efficacy of these medications has been substantiated by Kahng *et al*. [6]. That study reported that most patients attained normal hemoglobin levels by 8 months post-transplant and early iron therapy reduced the incidence of anemia, especially in females.

The primary goal of the current study was to assess the prevalence and management of anemia in a group of Chinese kidney transplant recipients. Therefore, whether the routine use of iron therapy for patients at risk of anemia can effectively prevent its occurrence without increasing other morbidities is still unclear. Further prospective studies are required. According to Mrug *et al*. [7], the quality of life and performance remarkably improves with treatment for anemia. Erythropoietin is seldom used in the management of PTA, but for patients with poor graft function and moderate to severe anemia, erythropoietin is most likely the only efficacious medication available.

ACEI and ARB have been successfully used to treat post-transplant erythrocytosis. Recent evidence suggests that the angiotensin II axis plays an important role in erythropoiesis [8], leading to the speculation that perhaps ACEI or ARB increases the chance of anemia in kidney transplant recipients. Other studies have suggested that azathioprine can cause anemia in the presence of concomitant ACEI use [8]. However, we observed no correlation between ACEI and anemia either administered with MMF or azathioprine in the current study. This may be partly attributed to the small number of participants in this study. In addition, a recent study suggests the renal benefits of losartan in kidney transplant recipients by reducing urinary excretion of proteins associated with tubular damage and graft fibrosis [9]. Taken together, the use of ACEI or ARB in kidney transplant recipients might be carefully balanced against the level of concern of PTA.

Retrospective studies, for their own limitations, can hardly provide accurate insight into the relative contribution of any single or combined immunosuppressive therapy in the development of anemia, and their shortcomings become even more overt when changes in immunosuppressive therapy have been made.

Previous studies have suggested that both MMF and azathioprine could lead to anemia [10]. The question then becomes which one is more likely to do so. In the current study, there was no significant difference in prevalence of anemia between patients receiving MMF and azathioprine at each year post-transplant. This result aligned with two other pivotal trials where a similar incidence of anemia between patients on these drugs were reported [11,12]. Kahng *et al*. [6] reported that patients on a dual therapy of MMF and prednisone had the lowest hemoglobin levels, and patients not being treated with MMF generally showed higher hemoglobin levels. However, claiming MMF is worse with regard to the occurrence of anemia is over-simplistic because many patients convert to MMF after a decline in kidney graft function is discovered, which can greatly confound the analysis. Studies assessing kidney transplant recipients who convert to MMF from azathioprine with good graft function may be in a better position to provide more precise information; however, no such comparisons have been made [13-15].

## Conclusions

PTA is a common complication for long-term follow-up kidney transplant recipients, and is often overlooked by transplant physicians. Kidney graft function is associated with both the diagnosis of anemia and hemoglobin levels. Iron medications should be administered in a timely manner in patients with PTA, while erythropoietin therapy should be initiated in anemic patients whose kidney grafts have already failed to produce sufficient erythropoietin to maintain hemoglobin levels.

## Abbreviations

ACEI: Angiotensin-converting-enzyme inhibitors; ANOVA: Analysis of variance; ARB: Angiotensin receptor blockers; BUN: Blood urea nitrogen; Ccr: Calculated creatinine clearance; MMF: Mycophenolate mofetil; PTA: Post-transplant anemia; Scr: Serum creatinine.

## Competing interests

The authors declare that they have no competing interests.

## Authors’ contributions

ZW performed the study, collected and analyzed the data, and wrote the paper. JG performed the study and wrote the paper. LL analyzed the data and wrote the paper. WW and SY performed the study. JT designed the study. JT takes responsibility as the guarantor for the content of the article. All authors read and approved the final manuscript.
